# Compartmentation of glycogen metabolism revealed from ^13^C isotopologue distributions

**DOI:** 10.1186/1752-0509-5-175

**Published:** 2011-10-28

**Authors:** Igor Marin de Mas, Vitaly A Selivanov, Silvia Marin, Josep Roca, Matej Orešič, Loranne Agius, Marta Cascante

**Affiliations:** 1 Department of Biochemistry and Molecular Biology, Faculty of Biology, Universitat de Barcelona, Av Diagonal 643, 08028 Barcelona, Spain; 2 Institute of Biomedicine of Universitat de Barcelona (IBUB) and CSIC-Associated Unit, Spain; 3 A.N.Belozersky Institute of Physico-Chemical Biology, MSU, Moscow 199899, Russia; 4 Hospital Clínic, IDIBAPS, CIBERES; Universitat de Barcelona, Barcelona 08028, Spain; 5 Technical Research Centre of Finland, Espoo, and Institute for Molecular Medicine, Helsinki, Finland; 6 Institute of Cellular Medicine, The Medical School, Newcastle University, Newcastle, UK

## Abstract

**Background:**

Stable isotope tracers are used to assess metabolic flux profiles in living cells. The existing methods of measurement average out the isotopic isomer distribution in metabolites throughout the cell, whereas the knowledge of compartmental organization of analyzed pathways is crucial for the evaluation of true fluxes. That is why we accepted a challenge to create a software tool that allows deciphering the compartmentation of metabolites based on the analysis of average isotopic isomer distribution.

**Results:**

The software Isodyn, which simulates the dynamics of isotopic isomer distribution in central metabolic pathways, was supplemented by algorithms facilitating the transition between various analyzed metabolic schemes, and by the tools for model discrimination. It simulated ^13^C isotope distributions in glucose, lactate, glutamate and glycogen, measured by mass spectrometry after incubation of hepatocytes in the presence of only labeled glucose or glucose and lactate together (with label either in glucose or lactate). The simulations assumed either a single intracellular hexose phosphate pool, or also channeling of hexose phosphates resulting in a different isotopic composition of glycogen. Model discrimination test was applied to check the consistency of both models with experimental data. Metabolic flux profiles, evaluated with the accepted model that assumes channeling, revealed the range of changes in metabolic fluxes in liver cells.

**Conclusions:**

The analysis of compartmentation of metabolic networks based on the measured ^13^C distribution was included in Isodyn as a routine procedure. The advantage of this implementation is that, being a part of evaluation of metabolic fluxes, it does not require additional experiments to study metabolic compartmentation. The analysis of experimental data revealed that the distribution of measured ^13^C-labeled glucose metabolites is inconsistent with the idea of perfect mixing of hexose phosphates in cytosol. In contrast, the observed distribution indicates the presence of a separate pool of hexose phosphates that is channeled towards glycogen synthesis.

## Background

^13^C isotope tracing, aimed in the evaluation of metabolic fluxes in living cells has been developing during last decades [1]. This experimental technique required a specific mathematical analysis, and it was created [2]. Currently, the stable isotope tracing of metabolites has been refined and is used to identify the adaptive changes of fluxes in man in normal and diseased states [3], in isolated cells [4], cancer cell cultures [5], and organisms such as fungi [6], yeast [7,8], etc. ^13^C tracer fluxomics can be combined with the analysis of gene and protein expressions to provide insight into multilevel regulation of cellular processes [9].

However, the rapidly developing experimental ^13^C tracer metabolomics surpasses the theoretical analysis of measured data. For a long time the detailed analysis of isotopomer distribution was possible only for isotopic steady state [10]. The tools applicable for analysis of non-steady state conditions appeared relatively recently [11-14], and the methodology of rule-based modeling used in some of these tools expanded to different areas of analysis of complex biological systems [15]. Although the analysis of ^13^C tracer data could result in the discovery of unknown metabolic pathways [16], the existing tools were designed mainly for the evaluation of metabolic fluxes assuming certain established topology of reaction network. However, ignoring the specificity of topology of particular reaction network, or in other words its compartmental structure, can compromise the results of metabolic flux analysis [17].

The topology of metabolic network could be complicated by substrate channeling [18-24], which could be seen as metabolite compartmentation. The latter follows from the definition, which says that a pathway intermediate is 'channeled' when, a product just produced in the pathway has a higher probability of being a substrate for the next enzyme in the same pathway, compared to a molecule of the same species produced in a different place [23,25].

Usually, studies designed for the analysis of channeling require invasive experiments, such as permeabilization of cells and determination of diffusion of labeled metabolites from or into the presumable channel [22-24]. However, it can be expected that experimental procedures destroy some kinds of channeling that occur in intact cells. Moreover, one cannot exclude the possibility that the metabolic channeling and compartmentation differ between various tissues and this could increase indefinitely the number of experiments necessary for defining the structure of metabolism in cells. Here, we propose a solution for such a problem: to determine the metabolic compartmentation by analyzing ^13^C isotopic isomer distributions in products of metabolism of labeled substrates; i.e. in the same study, which is designed for the evaluation of metabolic flux profile, thereby, not recruiting additional experiments.

Thus, the objective of the presented work is to create and implement a tool assessing the compartmentation based on ^13^C distribution. The challenge here is that, although the same compound, located in different subcellular spaces, likely possesses compartment-specific ^13^C signatures, the measurements average out the compartment-specificity [17,26,27]. The tool must help decipher the compartment-specific distribution of metabolic fluxes, consistent with the measured average labeling. Such deciphering is based on a simple idea that the compartment-specific simulation better fit ^13^C data, if the really existing compartments are taken into account. To estimate the goodness of data fit by various schemes of metabolic compartmentation we implement model discrimination analysis.

Two out of three experiments analyzed were described elsewhere [28], and metabolic fluxes were evaluated based on the application of simple formulas directly to experimental data. Such simple analysis (the only achievable then) does not account for all possible exchange of isotopes and their recycling. Moreover, these formulas imply that the network topology is known and can only give a formal ratio of main fluxes without its verification. Whereas the simulation of isotopic isomer distribution using the predicted fluxes can be compared with experiments and thus verify the predictions. Here, we describe the use of such simulations for the analysis of network topology, which is absolutely impossible by using simple formulas. The new analytical tool provides the opportunity to re-evaluate previously generated experimental data gaining new insights into the topology of the studied metabolic network, and assessing metabolic flux profile in detail in various physiological and pathological conditions.

## Results

### Accounting for channeling in the reaction scheme of model

**Figure 1:**
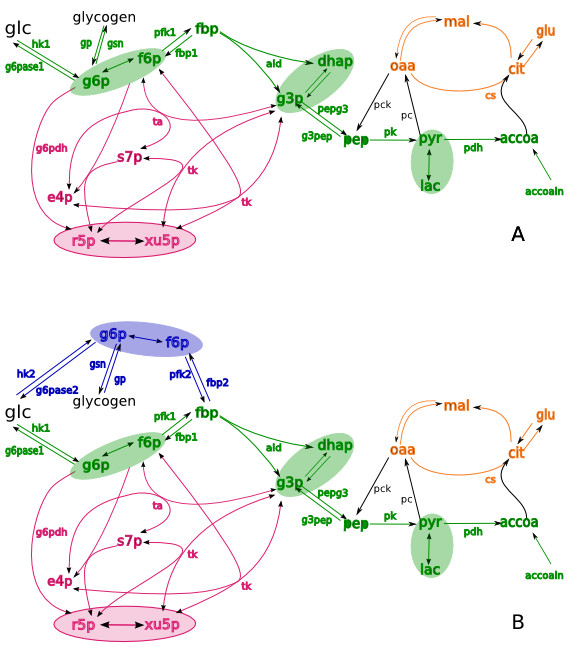
**The schemes of kinetic models used as a base for simulation of isotopologue distribution**. Metabolites are connected by biochemical reactions represented by arrows. Various colors indicate metabolites and reactions of specific pathways: green, glycolysis/gluconeogenesis; red, pentose phosphate pathways; orange, TCA cycle. The metabolites enclosed in ellipses are considered to be in fast equilibrium. (A), the basic model that includes one pool of hexose phosphates common for glycolysis and gluconeogenesis. (B), the model that includes also the additional pool of hexose phosphates (blue) that represents channeling in gluconeogenesis. Abbreviations are explained in the list of abbreviations in the text.

The dynamics of all possible isotopic isomers in glucose, lactate and glutamate from the incubation medium and glucose from glycogen in cell pellets, accumulated by two hours of incubation of liver cells with [1,2-^13^C_2_]D-glucose [28], were simulated with Isodyn using two different schemes that included either one or two pools of hexose phosphates, as shown in Figure 1. The conditions of incubation do not assume to reach steady state for the labeling of measured external metabolites, and the dynamic simulations correspond to non-steady state conditions of the experiment. The model, which does not consider channeling (referred to as model A), accounts for a single, well-mixed, common hexose 6-phosphate pool (Figure 1A). In accordance with the definition given in introduction, channeling assumes the existence of metabolite compartments, which could have had a different isotopomer (and isotopologue) composition and does not freely mix by diffusion with a pool of the same metabolite outside the channel. The presence of two compartments with different isotopomer compositions indicates metabolite channeling (Figure 1B), and the respective model is referred to as model B.

### Fitting the measured isotopologue distribution

The same stochastic algorithm of minimization of normalized deviations between the measured and computed data (χ^2^) in the global space of parameters (described in Methods) was applied to each model for data fitting. The fractions of isotopologues measured in glucose, lactate, fragments of glutamate and glycogen, and their best fit using the two models are shown in Table 1. χ^2 ^is shown for each metabolite separately and Σ_1_χ^2 ^sums all the individual χ^2^.

**Table 1:** Measured and simulated fractions of isotopologues and total concentrations of metabolites.

	Experiment		Simulated
	mean sd	Channeling	Mixed
**Glucose**		**χ^2^ = 0.442**	**0.406**
m0	0.512 ± 0.0069	0.511	0.511
m1	0.00913 ± 0.002	0.0084	0.00839
m2	0.478 ± 0.00652	0.481	0.481
[mM]	19.7 ± 1.92	20.4	20.2
**Lactate**		**χ^2^ = 1.43**	**7.32**
m0	0.86 ± 0.0482	0.839	**0.81**
m1	0.0235 ± 0.00802	0.0237	**0.0178**
m2	0.0946 ± 0.0388	**0.133**	**0.17**
m3	0.022 ± 0.0438	0.00381	**0.00145**
[mM]	0.81 ± 0.51	0.959	**1.48**
**Glutamate C2-C5**		**χ^2^ = 0.0564**	**0.0424**
m0	0.912 ± 0.0343	0.912	0.912
m1	0.0299 ± 0.0116	0.0301	0.0298
m2	0.0523 ± 0.0217	0.0574	0.0567
**Glutamate C2-C4**		**χ^2^ = 0.0049**	**0.00437**
m0	0.919 ± 0.0339	0.919	0.919
m1	0.0365 ± 0.00175	0.0356	0.355
m2	0.0446 ± 0.0166	0.0454	0.0451
**Glycogen**		**χ^2^ = 1.2**	**30.5**
m0	0.608 ± 0.0388	0.598	**0.658**
m1	0.0162 ± 0.0033	0.0151	**0.0271**
m2	0.362 ± 0.0351	0.375	**0.299**
m3	0.00399 ± 0.0011	0.00422	**0.00791**
m4	0.00961 ± 0.0026	0.00748	0.00749
m5	0.000464 ± 0.00016	0.000432	0.000533
mg/mL	0.355 ± 0.112	0.313	**0.232**
**Σ_1_ χ^2^**		**3.13**	**38.28**
**Glycogen C1-C4**		**χ^2^ = 1.42**	**6.68**
m0	0.613 ± 0.0448	0.627	**0.679**
m1	0.0224 ± 0.00834	**0.0133**	0.0297
m2	0.357 ± 0.0425	0.358	**0.289**
**Glycogen C3-C6**		**χ^2^ = 7.97**	**30**
m0	0.952 ± 0.00767	0.952	0.951
m1	0.00743 ± 0.00211	**0.0131**	**0.018**
m2	0.0371 ± 0.00467	0.0333	**0.0279**
**Σ_2_χ^2^**		**9.21**	**36.68**
**Σ_t_χ^2^ = Σ_1_χ^2^+Σ_2_χ^2^**		**12.52**	**74.95**

Isotopologues (m0, non-labeled; m1, containing one 13C isotope; m2, two 13C isotopes, etc) produced by isolated hepatocytes from glucose as the only substrate contained 50% of [1,2-13C2]D-glucose were measured in glucose from medium, glucose from glycogen and its fragments, lactate, and fragments of glutamate after two hours of incubation. The measurements are presented as mean ± standard deviation. The data were simulated using two models that either accounted for channeling or suggested a single "mixed" pool of hexose phosphates in accordance with the schemes presented in Figure 1. The fitting was performed using a stochastic algorithm described in Methods. The difference between the best fit and experimental data (χ2, see Methods) are shown for each metabolite and summarized for the whole set of data.

Model A that does not assume metabolite channeling in glycogen synthesis fitted the experimental isotopologue distribution with Σ_1_χ^2 ^= 38.28 (Table 1). 22 experimental points and 16 essential parameters, calculated as described in Methods, defined the number of degrees of freedom, F = 6. The given values of Σ_1_χ^2 ^and the number of degrees of freedom defining an extremely low value of incomplete gamma function Q = 9.9·10^-7 ^unambiguously indicates that the model which does not account for channeling should be rejected [29].

Conversely, model B that assumes channeling fits the measured isotopologue distribution much better, with Σ_1_χ^2 ^= 3.13 (Table 1). This model has four degrees of freedom, deduced from the same number of experimental points, and 18 essential parameters. These values defined Q = 0.536, which allowed the acceptance of the model.

Thus, the comparative study of two schemes based on the goodness of fit of the experimental data allowed rejection of the model that assumes a single common pool of hexose phosphates and acceptance of the alternative model, which accounts for channeling of intermediates in glucose metabolism.

### Model validation

Electron impact ionization used in mass spectrometry often splits molecules into fragments. Since the localization of such fragments in the molecule is known, the fact that a ^13^C atom belongs to a given fragment restricts the possible positions of this isotope in the molecule. This information can further restrict the possible set of solutions. The fractions of isotopologues from glycogen were measured not only in whole glucose molecules, but also in their fragments containing carbon atoms either 1-4 [28] or 3-6. For model B, the best fit described above fits also the whole set of data including the fragments of glucose from glycogen as Table 1 shows, giving Σ_t_χ^2 ^= 12.52. This value and 8 degrees of freedom deduced for this model from 29 experimental points and 21 essential parameters define the value Q = 0.129. This value indicates that the model is acceptable, thus confirming the conclusion based on the simulation of ^13^C distribution in the whole molecule of glucose from glycogen without accounting for the fragments.

As Table 1 shows, model A fits the whole set of data with Σ_t_χ^2 ^= 74.9. With number of degrees of freedom of 11 (29 experimental points and 18 essential parameters), the value of Q was 1.42·10^-11^. This value further indicates that the model of a homogeneous pool of hexose phosphates should be rejected.

Another validation of the channeling came from a series of two experiments where hepatocytes were incubated in the presence of glucose and lactate (as described in Methods). The conditions in the two experiments were virtually identical with the exception that glucose was labeled in one of these experiments [28] and lactate in the other. Cells consumed lactate, and the label from lactate ascended up to extracellular glucose as shown in Table 2. In these two experiments, the incubations with liver cells resulted in different isotopic isomer distribution since different substrates were labeled at the beginning. However, the same conditions for cell metabolism suggest that the same set of metabolic fluxes must fit the isotopologue distribution measured in both the experiments. Respectively, the algorithm subsequently performed the simulations of two experiments for the same set of model parameters and summed χ2 for both simulations. The algorithm for parameter optimization had searched for the set of parameters that minimized Σχ^2 ^and Table 2 shows the best fit of the two experiments performed using each of the two models.

**Table 2:** Isotopologue distribution produced by isolated hepatocytes in the presence of glucose and lactate.

	Experiment 1	Simulations		Experiment 2	Simulations	
	label in glucose	B	A	label in lactate	B	A
**glucose:**		**χ^2^ = 7.21**	**5.72**		**χ^2^ = 5.52**	**0.576**
m0	0.532 ± 0.0098	0.514	0.514	0.979 ± 0.01	0.976	0.984
m1	0.00846 ± 0.0022	0.0114	0.00918	0.0063 ± 0.0055	0.00484	0.00354
m2	0.459 ± 0.0103	0.473	0.475	0.0055 ± 0.0064	0.00891	0.00469
m3	-- --	--	--	0.0064 ± 0.0016	0.00996	0.00628
[mM]	20.6 ± 2.91	21	21.1	20.9 ± 2.22	20.8	20.9
**glycogen:**		**χ^2^ = 6.46**	**27.3**		**χ^2^ = 2.22**	**5.73**
m0	0.681 ± 0.032	0.683	0.57	0.909 ± 0.026	0.907	0.9
m1	0.0119 ± 0.031	0.00767	0.0131	0.017 ± 0.0066	0.0166	0.0225
m2	0.302 ± 0.031	0.308	0.409	0.038 ± 0.01	0.0313	0.0298
m3	0.0017 ± 0.001	0.000136	0.00208	0.0273 ± 0.0071	0.0363	0.04
m4	0.0032 ± 0.0016	0.000593	0.00565	0.0036 ± 0.0016	0.00329	0.00317
m5	-- --	--	--	0.003 ± 0.014	0.00335	0.00271
mg/mL	0.263 ± 0.084	0.256	0.196	0.262 ± 0.0691	0.256	0.196
**glgn14:**		**χ^2^ = 5.31**	**19.2**		**χ^2^ = 2.23**	**4.59**
m0	0.678 ± 0.032	0.692	0.588	0.93 ± 0.024	0.924	0.921
m1	0.016 ± 0.0046	0.00561	0.0112	0.033 ± 0.009	0.0343	0.0472
m2	0.3 ± 0.032	0.302	0.399	0.019 ± 0.0079	0.0178	0.0128
m3	-- --	--	--	0.014 ± 0.005	0.0209	0.0163
m4	-- --	--	--	0.004 ± 0.003	0.00267	0.0264
**glgn36:**		**χ^2^ = 1.48**	**14.2**		**χ^2^ = 1.97**	**4.48**
m0	0.98 ± 0.0101	0.987	0.968	0.924 ± 0.024	0.923	0.911
m1	0.00408 ± 0.0018	0.0051	0.0101	0.0265 ± 0.007	0.0332	0.0348
m2	0.0139 ± 0.0078	0.00766	0.216	0.027 ± 0.0081	0.0188	0.0219
m3	-- --	--	--	0.021 ± 0.0067	0.0221	0.0292
**lactate:**		**χ^2^ = 2.06**	**2.73**		**χ^2^ = 1.62**	**9.76**
m0	0.974 ± 0.026	0.991	0.985	0.636 ± 0.017	0.621	0.608
m1	0.0026 ± 0.0019	0.000974	0.00135	0.0166 ± 0.0025	0.0167	0.0172
m2	0.0094 ± 0.0037	0.00773	0.0141	0.0318 ± 0.0035	0.0302	0.0245
m3	0.00136 ± 0.023	0.00000227	0.0000436	0.316 ± 0.0213	0.332	0.35
[mM]	6.18 ± 0.75	6.8	6.73	3.18 ± 0.43	6.29	6.22
**Σχ^2^**		**22.52**	**69.15**		**13.56**	**25.136**

Before incubation the medium contained either 50% of [1,2-13C2]D-glucose and unlabeled lactate (experiment 1) or 50% uniformly 13C-labeled lactate and unlabeled glucose (experiment 2). The measurements are presented as mean ± standard deviation. The data were fit by two models (A and B). The conditions of incubation and measurements, and data fitting are described in Methods. The difference between the best fit and experimental data (χ2, see Methods) are shown for each metabolite and summarized for the whole set of data.

Model B fits both the experiments with Σχ^2 ^= 36.1 (22.52 when label is in glucose and 13.56 when label is in lactate). Both the experiments together provided 46 points. With 24 essential parameters, this problem has 22 degrees of freedom that corresponds to Q = 0.09, thus indicating that the model is acceptable. If this set of parameters which gives the best fit for model B is used for model A (where the same flux of glycogen synthesis is directed from the common pool of hexoses), Σχ^2 ^increases up to 331, where glycogen gave the greatest Σχ^2^. The fitting procedure reduced Σχ^2 ^for model A down to 94.286 (69.15 when label was in glucose and 25.13 when label was in lactate) with the isotopologue distribution shown in Table 2. For this best fit, the same number of experimental points (46), and 24 essential parameters (22 degrees of freedom) give Q = 6.325·10^-11^, which indicates that model A is incorrect.

### Metabolic flux distribution

If model discrimination analysis indicates that a model should be rejected, as in the case of model A, the distribution of metabolic fluxes obtained with such a model cannot be reliable. In contrary, if the analysis suggests accepting a model, as in the case of model B, there is much more confidence that it evaluates true metabolic fluxes. Therefore we analyzed the distribution of metabolite fluxes computed with model B. Table 3 shows metabolic fluxes corresponding to the best fit of the measured data by model B, together with their 99% confidence intervals. The metabolic fluxes of the two experiments, performed in the presence of lactate using two different substrate labelings, were the same (Table 2). Thus, Table 3 shows two sets of fluxes with their confidence intervals evaluated by model B. One of the sets corresponds to the best fit of isotopologue distribution measured only in the presence of glucose (as shown in Table 1), and the other in the presence of glucose and lactate (as shown in Table 2).

**Table 3:** Metabolic fluxes corresponding to the best fit of experimental data and their 99% confidence intervals

	Glucose as the only substrate	Glucose with lactate	
	99% confidence interval	99% confidence interval	
	bestfit (min - max)	bestfit (min - max)	Model A
**hk1**	0.0026894(0.002150-0.003080)	0.152266(0.074815-0.377413)	0.01981
**hk2**	0.0021436(0.001710-0.002480)	0.0056939(0.0041563-0.0076078)	
**g6pase1**	5.50E-05(2.70E-5-7.80E-5)	0.150611(0.073125-0.375565)	0.01809
**g6pase2**	2.13E-05(0.0-0.000066)	0.005166(0.0029064-0.0071982)	
**pfk1**	0.003048(0.002370-0.003530)	0.0023558(0.0015063-0.0046942)	0.00345
**pfk2**	0.0004816(0.0-0.000950)	0.0007975(0.0006197-0.0012778)	
**fbpase1**	0.0004446(0.000270-0.000560)	0.0013469(0.0005781-0.0023709)	0.00363
**fbpase2**	0.0005496(0.000330-0.000690)	0.0023329(0.00161-0.0032427)	
**gp**	2.56E-06(1.70E-6-5.10E-6)	0.000143(5.12E-05-0.0001883)	0.00000
**gs**	0.0021929(0.001660-0.002680)	0.0022087(0.0018593-0.0027097)	0.00188
**aldf**	0.0192413(0.007480-0.022580)	0.0159083(0.0153142-0.0163931)	0.01608
**aldr**	0.016706(0.004480-0.020410)	0.0164349(0.0153054-0.016708)	0.01625
**aldex**	0.0424386(0.010420-0.056150)	0.0137425(0.0103922-0.0195718)	0.01565
**g3pep**	0.0064103(0.004490-0.007700)	0.258278(0.1677705-0.3394195)	0.12224
**pepg3**	0.0013391(0.000140-0.002370)	0.258701(0.166264-0.339572)	0.12257
**pk**	0.0050704(0.003910-0.006230)	0.0199774(0.0157079-0.0239355)	0.01497
**lacin**	1.71E-07(1.20E-7-2.60E-7)	0.231716(0.1714965-0.2686475)	0.12266
**lacout**	0.00507(0.003910-0.006230)	0.222326(0.1621185-0.2590095)	0.11074
**pc**	1.20E-07(5.70E-8-1.90E-7)	0.0204457(0.0155337-0.0233495)	0.01542
**pepck**	1.16E-08(5.30E-9-2.80E-8)	0.020401(0.0154933-0.0232863)	0.01530
**maloa**	1.85E-07(9.60E-8-3.00E-7)	0.0835934(0.048937-0.111892)	0.03396
**oamal**	3.80E-08(1.30E-8-8.10E-8)	0.0747156(0.0368199-0.0997713)	0.02261
**cs**	2.55E-07(1.30E-7-4.00E-7)	0.008922(0.0069631-0.0147641)	0.01147
**citmal**	1.47E-07(8.40E-8-2.30E-7)	0.0088778(0.0069227-0.0146991)	0.01135
**pdh**	2.75E-07(7.70E-8-5.00E-7)	0.0089212(0.0069623-0.0147633)	0.01147
**g6pdh**	3.87E-06(2.80E-6-7.50E-6)	0.0018986(0.0013506-0.0021492)	0.00000
**p5p > s7p**	0.0011849(0.000880-0.002270)	0.0006229(0.0004459-0.0007215)	0.00051
**s7p > r5p**	0.0011925(0.000890-0.002280)	4.35E-06(2.56E-06-2.98E-05)	0.00047
**f6p > p5p**	9.59E-06(4.20E-6-3.40E-5)	4.02E-05(1.17E-05-9.84E-05)	0.00000
**p5p > f6p**	4.93E-06(2.80E-6-2.80E-5)	0.0006793(0.0004937-0.000751)	0.00000
**f6p > s7p**	1.85E-05(9.20E-6-7.30E-5)	1.40E-05(4.56E-06-2.45E-05)	0.00000
**s7p > f6p**	9.56E-06(4.80E-6-6.60E-5)	1.65E-06(9.03E-07-8.56E-06)	0.00000
**p5p-g3p**	0.0006152(2.70E-4-2.83E-3)	0.0017868(0.0007706-0.0033249)	0.00036
**f6p-s7p**	7.69E-08(2.10E-8-7.70E-7)	1.53E-05(4.62E-06-2.78E-05)	0.00000
**p5p-s7p**	0.0022967(1.37E-3-7.06E-3)	1.51E-06(8.37E-07-8.16E-06)	0.00067
**f6p > s7p**	0.0015144(0.000850-0.001940)	0.000862(0.0002796-0.0021263)	0.00117
**s7p-f6p**	0.0015015(0.000850-0.001920)	0.0014894(0.0008194-0.0026708)	0.00119
**f6p-g3p**	0.0075338(0.002170-0.013330)	0.0164459(0.0085357-0.0352201)	0.00230
**s7p-e4p**	0.0003018(0.000140-0.000550)	7.81E-05(2.01E-05-0.0002102)	0.00061

The names of fluxes are given in the list of abbreviations in the text.

At first glance, a notable difference is seen between metabolic fluxes under the two different conditions. The fluxes for the best fit indicate that the presence of lactate had perturbed the entire central carbohydrate metabolism of hepatocytes. Without lactate, almost half of the glucose consumed(hk) was used to replenish the glycogen store (glgsn) that was exhausted during starvation, and the rest was mainly converted to lactate except a small part that was burned in TCA cycle. Although net consumption of glucose did not change much by the addition of lactate, the fluxes of glucose input (hk) and output (g6ph) taken separately are increased by almost two orders of magnitude. Thus, recycling of metabolites increased without affecting the net influx of glucose. The addition of lactate increased recycling in many other points downstream of glucose entrance. This refers to the flux through fructose bisphosphatase (fbpase), which forms a futile cycle with phosphofructokinase (pfk). The increase of flux transforming glyceraldehyde 3-phosphate into phosphoenolpyruvate (g3pep, it lumped a set of reactions) is accompanied by the increase of reactions in the reverse direction (pepg3). Essentially there is an increased futile recycling through pyruvate kinase (pk), pyruvate carboxylase (pc) and phosphoenolpyruvate carboxykinase (pepck). This recycling is accompanied by an increase of flux through the TCA cycle (pdh, cs, citmal) linked with enhancing of energy production. Some changes took place in the pentose phosphate pathway, but they were not as impressive as in glycolysis and TCA cycle.

The confidence intervals for some of the fluxes (e.g. hkI) were large. However, many intervals for the two studied conditions do not overlap, and so the changes described above for the best fit remain qualitatively the same for the whole intervals.

For each fit (as it can be seen in best fit fluxes presented in Table 3) the ratio of the two inputs is different for the two hexose phosphate pools. The hkII is less than hkI, and, in contrary, the contribution of fbpaseII is higher than fbpaseI. This results in a different isotopomer distribution in glycogen relative to the glucose in the medium. Moreover, the reactions of the pentose phosphate pathway that interchange various sugar phosphates with fructose phosphates introduce additional differences to the isotopomer content between glycogen and hexose phosphates fueling glycolysis.

Among the fluxes of pentose phosphate pathway, the most essential are the exchange between triose and pentose phosphates, and fructose and sedoheptulose phosphates. These exchanges also contribute to the difference between isotopomer content of glycogen and hexose phosphates fueling glycolysis.

Although model A was rejected, the fluxes corresponding to the best fit in the presence of lactate are shown in the last column of Table 3. They are different from those computed with model B. This difference shows the possible error in the results if the applied model does not properly account for the compartmentation of metabolites.

## Discussion

### Possible sources of errors and the implemented way of avoiding them

Stable isotope tracing is a promising sensitive technique for the study of metabolism in living cells. However, it is sensitive to various flaws and incompleteness in the data analysis. That is why tools for tracer data analysis must take into account the possible sources of errors. In particular, omitting some isotope exchange reactions may lead to significant errors in the calculated flux distributions [17]. The model used here simplifies some sequential reactions of the pentose phosphate pathways, TCA cycle, and glycolysis, which are grouped together. However, all the possible splitting and re-formations of carbon skeleton in the considered pathways are taken into account [30,31]. This gives a confidence in avoiding potential errors related with the simulation of incomplete set of fluxes.

Not accounting for compartmentation due to metabolic channeling is another pitfall that can result in incorrect estimation of metabolic fluxes [17]. Table 3 shows that model A, which does not account for channeling, gives remarkably different results. However, based on the current state of the art in fluxomics, it is difficult to include into consideration all the possible microcompartments *a priori*. Although there are studies that confirm the associations of enzymes and channeling [18-24], it is not clear how general the studied cases are with regard to various organisms and tissues, and whether the metabolite compartmentation studied *in vitro *still persist *in vivo*. In modeling the metabolic networks, considering a single well mixed pool for each metabolite still remains commonly accepted (e.g. [32]). The method proposed here to determine metabolite compartmentation from ^13^C distribution in metabolites does not require specific experiments. Instead, it requires a specific analysis related with the implementation of various schemes and application of model discrimination analysis to define the compatibility of the schemes with the data.

### Experimental design facilitating the analysis

To restrict the possible ways of label propagation, the experimental system was simplified to a maximum by excluding the other sources of carbon except glucose or lactate. This permitted us to find that measured isotopologue distribution even in a small number of metabolites limits the possible solutions sufficiently to reject the hypothesis about unique well mixed pool of hexose phosphates.

The more metabolites analyzed, the more information about the topology of metabolic network they can potentially bring. To extract such information much more various hypothetic topologies must be analyzed. As an introduction to the isotopomer-based analysis of network topology, we presented a simple case of a few metabolites. However, the commencement from small dataset facilitates further extension of this method to larger datasets, including those obtained with NMR or MS [33-35].

### Channeling from the point of view of limited diffusion

Although it is known that from the point of view of ^13^C distribution channeling can be simulated as an additional compartment [14], this particular channeling in glycogen synthesis was never considered before and the presented work verified its consistency with experimental data. The analysis of channeling was based on the comparison of the models accounting for well-mixed versus a compartmentalized pool of hexose phosphates. The result of analysis was the rejection of hypothesis suggesting well-mixed in favor of compartmentalized pool of hexose phosphates. To form a separate pool of hexose phosphates and use it specifically for glycogen synthesis, all the three enzymes, hexokinase, phosphoglucomutase, and fructose bisphosphatase, must have access to it. This indicates that these enzymes are spatially co-localized within the intracellular compartment secluded from the rest of the cytosol by diffusion barriers. The indications that diffusion in biological structures can be essentially limited appear in various studies of cell physiology. The diffusion of cAMP is possibly extremely restricted in the vicinity of cyclic nucleotide-gated channels [36,37]. There are various indications that the diffusion of ATP is restricted in the proximity of K_ATP _channels [38-40] and in myofibrils [41]. Probably, the kind of compartmentation revealed here indicates the general situation in the cell: diffusion is highly limited by the bodies of macromolecules, which serve as borders of microcompartments for small metabolites.

### Matching schemes to the types of isotopomer distribution

The acceptance of model B does not mean that the set of parameters and respective fluxes can be defined unambiguously. The large confidence intervals for some fluxes (shown in Table 3) specify an ensemble of sets of metabolic fluxes that are consistent with the data. Such a situation is quite normal in biological data analysis [42-45], when models can make robust predictions with regards to the behavior of the studied system (in our case the distribution of isotopologues), but such prediction remains valid for various sets of parameters (fluxes). In such cases, either the topology of the model that is consistent with the data, or the prediction of system behavior for the accepted ensemble of parameter sets (expected change of isotopologue distribution with a change of conditions) can be the main result of the use of the model. In this way the analysis consists in mapping between the schemes of metabolism and specific distributions of isotopomers.

### Change of hepatocyte metabolism in the presence of lactate

Despite the large confidence intervals, the change of metabolic state in the presence of lactate is evident. Most of the metabolic fluxes increased so much that confidence intervals for them do not overlap with those found for glucose as the only substrate. Lactate induced the substantial increase of metabolite recycling. This result of modeling, in principle, agrees with the direct observation, that an essential amount of label from lactate ascends up to medium glucose, and an essential amount of label from glucose descends down to lactate. It is in accordance with the function of the liver, which can utilize lactate to synthesize glucose.

However, quantitatively, some of the results are not so evident. Table 3 shows that the recycling in upper glycolysis (between glucose and hexose-6-phosphates) increases several orders of magnitude in the presence of lactate. This recycling brings isotope composition of hexose-6-phosphates formed by gluconeogenesis into the pool of glucose, and *vice versa*, provides glucose isotopomer composition for the pool of hexose-6-phosphates, which are further split to trioses. Indeed, χ^2 ^criterion is very sensitive to the value of such recycling: its two-fold decrease leads to the χ^2 ^increase from 36.1 to 46.6 (data not shown), which indicates that the flux that decreased twice was out of 99.9% confidence interval for this recycling. The high velocity of this futile cycle results in the high rate of ATP consumption. However, it agrees in the order of magnitude with ATP production, taking into account that the flux through TCA cycle produces five folds more NADH (15 folds more ATP).

The net glucose consumed as well as lactate produced are burned through the TCA cycle thus producing energy necessary for the recycling. Thus, in the presence of only glucose, its essential part is used to replenish glycogen, whereas in the presence of both glucose and lactate the cultured liver cells apparently burns these substrates (preferentially lactate).

The presented analysis of the entire set of experiments characterized the capacity of hepatocytes to modify metabolic state under extreme conditions. The characterization of metabolism of hepatocytes is inseparable from the detection of the real compartmentation of considered pathways. Application of this methodology to larger datasets will reveal new information about the network topology. It opens a perspective to examine the compartmentation and metabolic flux profile in various cells under physiological and patho-physiological conditions.

## Conclusions

Compartmentation of intracellular metabolism, appeared as a general phenomenon, results that the analysis of metabolic flux distribution should be inseparable from the analysis of compartmental structure of studied pathways. Here we proposed a methodology implemented in our software to reveal compartmental structure and metabolic flux distribution from the distribution of ^13^C isotopomers measured in the products of cells incubated with ^13^C labeled substrates. This methodology is based on varying the schemes for simulation of measured data and applying the model discrimination analysis. The application of this methodology to the analysis of ^13^C isotopomer distributions measured in metabolites of isolated liver cells revealed a separate compartment of hexose phosphates related with substrate channeling in glycogen metabolism. This analysis provided the distribution of metabolic fluxes in central carbohydrate metabolism of the cells incubated with ^13^C labeled glucose, and revealed the changes of fluxes that were induced by addition of lactate in the incubation media.

## Methods

To analyze cellular metabolic flux profiles for specific conditions *in situ *and various schemes of metabolic reactions we used the software tool "Isodyn" (from "isotopomer dynamics") [11,12]. It simulates isotopomer distributions in the same way as classical kinetic models simulate the time-course of metabolite concentrations.

### Models and data fitting

The systems of differential equations corresponding to the schemes presented in Figure 1 and expressions for the rates of individual reactions are given in Additional File 1. The metabolite fluxes and concentrations were obtained as a numerical solution of the differential equations using the BDF method implemented in our software Isodyn. The software then uses these values of total concentrations and fluxes to construct and solve differential equations for all isotopomers of metabolites presented in Figure 1. The algorithms for constructing equations for isotopomers are described elsewhere [11,12].

The concentrations of isotopologues needed to be compared with experimental data were calculated as a sum of the respective isotopomer concentrations computed by Isodyn. Fitting of the experimental data was performed by minimizing χ^2^, the square of deviations between measured isotopologue fractions (y_i_) and values (y(x_i_, a)) computed for the set of parameters, a, as fractions of isotopologues x_i_, normalized by experimental standard deviations (σ_i_):

$$\chi^{\text{2}}=\overset{N}{\underset{i=\text{1}}{\sum }}{\left[\frac{y_{i}-y\left(x_{i};a\right)}{\sigma_{i}}\right]}^{\text{2}}$$

The minimization was performed in the global space of parameters using our implementation of simulated annealing algorithm supplemented by coordinate descent in local area [5]. The sets of parameters, thus optimized, defined the sets of metabolic fluxes resulting in simulated isotopologue distributions and the value of *χ*^2^. Multiple application of the optimization resulted in multiple sets of fluxes characterized by different values of *χ*^2^. Application of *χ*^2^ threshold to the obtained sets of fluxes [29] defined confidence intervals for the metabolic fluxes. The implementation of this procedure in Isodyn was described elsewhere [5].

### Modification of reaction scheme

A change of equations of the basic kinetic model could be usually performed easily, even graphically: there are algorithms that transform a drawn scheme into a system of ordinary differential equations (ODE) [46]. However, in the case of Isodyn the removal or addition of reactions in the basic kinetic model must be followed by the respective changes in calculation of the isotopomer distribution. The consistent change of both the modules (basic kinetic model and calculation of respective isotopomer distributions) was performed automatically and the sources of respective programs are available for free at http://www.bq.ub.es/bioqint/label_distribution/tutorial.tar.gz..

### Aldolase reaction

An essential restriction, which helps to distinguish between models, is including the interdependency of fluxes catalyzed by the same enzyme, as we have shown for transketolase and transaldolase [12]. Here, similar interdependency of various isotope-exchange fluxes is considered for the aldolase-catalyzed reaction [30], which normally are not included in classical kinetic models.

**Figure 2:**
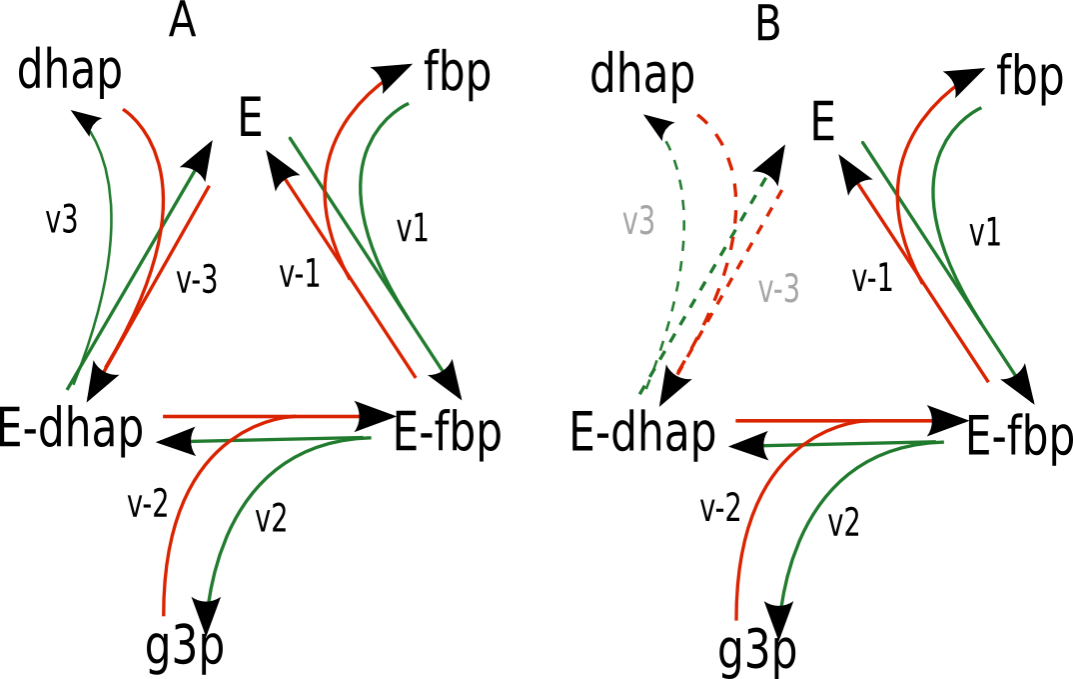
**Isotope-exchange fluxes in the aldolase-catalysed reaction**. **(A) **shows the whole reaction cycle when the enzyme (E) forms a complex with fbp (E-fbp), releases g3p keeping dhap (E-dhap), and finally releases dhap returning to (E). Forward flux (green lines) through the whole reaction cycle brings isotopes originated from the fbp pool into the pools of dhap and g3p, and reverse flux (red lines) brings isotopes originated from the pools of dhap and g3p into fbp pool. **(B) **shows forward (green lines) and reverse (red lines) fluxes that only exchange isotopes of upper part of fbp molecule with g3p pool without releasing dhap. v_i _designate the respective rates of elementary steps of reaction mechanism.

The scheme in Figure 2 shows the possible isotope-exchange fluxes in the aldolase reaction, accounted for in the model. The flux, shown in Figure 2A with green lines, transforms the whole molecule of fbp in the pool of trioses. It constitutes only a part of the flux v_3_, because another part of v_3_ produces dhap originated not from fbp, but from the same pool of trioses, bound to the enzyme through the reaction v_-3_. The steady state fraction of v_3_ that produces dhap originated from fbp (that equals to the fraction of bound dhap originated from fbp (PfE-dhap)) can be expressed as the ratio of input of molecules originated from fbp to the total input in E-dhap:

(1) $$P_{E-dhap}^{f}=\frac{v_{\text{2}}.P_{E-fbp}^{f}}{v_{\text{2}}+v_{-\text{3}}}$$

Here Pf_E-fbp_ is the fraction of v_2_, which brings the carbons originated from fbp to E-dhap (or the fraction of bound fbp originated from fbp). Another fraction of v_2_, (1-P^f^_E-fbp_) brings carbons originated from triose pool, which were bound through reactions v_-3 _and v_-2. _Pf_E-fbp_, in turn, can be expressed as the ratio of input of molecules originated from fbp to total input in E-fbp:

(2) $$P_{E-fbp}^{f}=\frac{v_{-\text{2}}.P_{E-dhap}^{f}+v_{\text{1}}}{v_{\text{1}}+v_{-\text{2}}}$$

The solution of equations (1) and (2) is

(3) $$P_{E-dhap}^{f}=\frac{v_{\text{2}}.v_{\text{1}}}{v_{\text{2}}.v_{\text{1}}+v_{-\text{3}}.v_{\text{1}}+v_{-\text{3}}.v_{-\text{2}}}$$

where the rates vi could be expressed through the rate constants and substrate concentrations. The forward flux through the whole cycle (indicated by green lines in Figure 2A) is

(4) $$v_{f}=P_{E-dhap}^{f}.v_{\text{3}}=\frac{v_{\text{1}}.v_{\text{2}}.v_{\text{3}}}{v_{\text{2}}.v_{\text{1}}+v_{-\text{3}}.v_{\text{1}}+v_{-\text{3}}.v_{-\text{2}}}$$

The reverse flux of fbp formation (red lines in Figure 2A) from triose phosphates (P^t^_E-fbp _) could be described similar to (4):

(5) $$v_{r}=P_{E-fbp}^{t}.v_{-\text{1}}=\frac{v_{-\text{1}}.v_{-\text{2}}.v_{-\text{3}}}{v_{\text{2}}.v_{\text{1}}+v_{-\text{3}}.v_{\text{1}}+v_{-\text{3}}.v_{-\text{2}}}$$

Figure 2B shows two additional fluxes, which only exchange half a molecule of fbp with g3p pool. As in the cases described above, the fraction of the first three carbons originated from fbp (P^fg^_E-dhap_) in (E-fbp) is:

(6) $$P_{E-fbp}^{fg}=\frac{v_{\text{1}}}{v_{\text{1}}+v_{-\text{2}}}$$

and the forward (thin black in Figure 2B) flux:

(7) $$v_{fg}=\frac{v_{\text{1}}.v_{\text{2}}}{v_{\text{1}}+v_{-\text{2}}}$$

The flux in the opposite direction (indicated by thick gray lines) is described likewise.

Thus, the model accounts for three isotope-exchange fluxes related with aldolase activity: forward and reverse flux through the whole cycle of enzyme reaction, and pure isotope exchange flux between f6p and g3p, without the change of total concentrations of these metabolites. They are not used in classical kinetic simulations, where only the net flux is important, but they are necessary for the subsequent simulation of isotopologue distribution. The isotope-exchange fluxes of transketolase and transaldolase were implemented similarly as described elsewhere [12].

### χ^2 ^criterion for the acceptance or rejection of model

To analyze the structure of metabolic networks, model discrimination analysis was used to test various kinetic models of the same pathways and reject the ones inconsistent with experimental data. Isodyn implements criteria for acceptance or rejection of a model based on the values of normalized square of difference between experimental data and simulation (χ^2^) and numbers of degrees of freedom [29].

The fitting algorithm implemented in Isodyn identifies the global minimum for the function χ^2 ^and the respective set of parameters and fluxes. If the model is acceptable, the estimated fluxes are also acceptable as a model prediction. The value of χ^2 ^is used in Isodyn as a criterion for acceptance or rejection of model as it is described in [29]. The probability that a model with F degrees of freedom is correct and χ^2 ^by chance could exceed a determined value, is given as an incomplete gamma function (Q(a,x), where a = F/2 (F is the number of degrees of freedom), and x = χ^2^/2):

(8) $$Q\left(a,x\right)=\frac{\text{1}}{\Gamma \left(a\right)}\overset{x}{\underset{\text{0}}{\int }}e^{-t}t^{a-\text{1}}dt$$

Here Γ(a) is gamma function:

(9) $$\Gamma \left(z\right)=\int t^{z-\text{1}}e^{-t}dt$$

The model is acceptable if Q value is larger than 0.05. It can be acceptable even with Q value larger than 0.001, if the errors are not normal or have been moderately underestimated. But if the Q value is lower than 0.001, the model must be rejected as inconsistent with experimental data.

### Estimation of number of degrees of freedom

Formally, the number of degrees of freedom (F) is calculated as the difference between number of data points (N, which in our case is the number of fractions of isotopologues for all measured metabolites and total metabolite concentrations) and parameters (P) in the model:

(10) F=N-P

However, in the case if the model is underdetermined, it could happen that the fit of the given data is insensitive to some parameters or there are ambiguous combinations equally affecting the fit, so that the parameters could not be distinguished. The presence of such parameters does not improve the fit and thus do not decrease the number of degrees of freedom. Both situations result in the fact that the Hessian matrix (the matrix of second derivatives of objective function *χ*^2^ with respect to $\left[\alpha_{kl}\right]=\left[\frac{\partial^{\text{2}}\chi^{\text{2}}}{\partial a_{k}\partial a_{l}}\right]$ sparameters,) is singular, or numerically close to singularity. In such situations the real number of degrees of freedom is higher than the formal difference (10). The maximal set of parameters that could be defined by the given experimental data could be estimated based on the analysis of Hessian matrix.

This matrix is calculated as follows. First derivative of χ^2^ with respect to the parameters is:

(11) $$\frac{\partial \chi^{\text{2}}}{\partial \alpha_{k}}=-\text{2}\overset{N}{\underset{i=\text{1}}{\sum }}\frac{\left[y_{i}-y\left(x_{i};a\right)\right]}{\sigma_{i}^{\text{2}}}\frac{\partial y\left(x_{i};a\right)}{\partial \alpha_{k}}$$

where k = 1,2,...M (number of parameters)

Differentiation of these functions gives

(12) $$\frac{\partial^{\text{2}}\chi^{\text{2}}}{\partial \alpha_{k}\partial \alpha_{l}}=-\text{2}\overset{N}{\underset{i=\text{1}}{\sum }}\frac{\text{1}}{\sigma_{i}^{\text{2}}}\left[a-b\right]$$

Where:

$$a=\frac{\partial y\left(x_{i};a\right)}{\partial \alpha_{k}}\frac{\partial y\left(x_{i};a\right)}{\partial \alpha_{l}}$$

$$b=\left[y_{i}-y\left(x_{i};a\right)\right]\frac{\partial^{\text{2}}y\left(x_{i};a\right)}{\partial \alpha_{k}\partial \alpha_{l}}$$

The second term under the sum, which contains the second derivative of fitting function y(xi,a) is usually ignored [27]:

(13) $$\alpha_{kl}=-\text{2}\overset{N}{\underset{i=\text{1}}{\sum }}\frac{\text{1}}{\sigma_{i}^{\text{2}}}\left[\frac{\partial y\left(x_{i};a\right)}{\partial a_{k}}\frac{\partial y\left(x_{i};a\right)}{\partial a_{l}}\right]$$

As it is indicated above, the singularity of this matrix means that some parameters of the model could not be defined in principle, given the specific dataset analyzed. To find out what characteristics of the studied system (parameters of the model) could be revealed, given the model and a specific dataset, the standard procedure of singular value decomposition of Hessian matrix was used [29].

Following this standard routine the Hessian matrix A is decomposed to the product of orthogonal matrix U, the vector (or diagonal matrix) of singular values W, and the orthogonal matrix V. The ratio of maximal and minimal values in the vector W, called condition number, characterizes the singularity of A. If some values of W are zeros, matrix A is strictly singular, but it could be numerically close to singularity, or ill-conditioned, if the condition number is close to machine precision. The covariance matrix C = A-1 was found from singular value decomposition as C = (V·W-1·UT), where W-1 = [diag(1/wi)]. Diagonal elements of C are the variances of parameters and the other elements are covariances. If matrix A is ill-conditioned, its inverse C cannot be defined and the failure of finding the inverse indicates that the number of parameters is excessive.

Isodyn finds the maximal set of parameters of the model, which, being considered as a subject for fitting, give a non-singular Hessian matrix. The size of this set could be considered as the number of parameters, which affects the number of degrees of freedom in the model with regard to given experimental data. If in fact the model has more parameters, the other parameters are not distinguishable by the given experimental data and must be considered as constants. The number of degrees of freedom (F) is defined as a difference between the numbers of experimental data and effective model parameters, and the value of incomplete gamma function Q(F/2, χ2/2), defined as described in [29], indicates the acceptance of the model.

## Experimental methods

### Materials

[1,2-^13^C_2_]D-glucose (> 99% enriched) and [U-^13^C_3_]L-lactate (> 99% enriched) were purchased from Isotec (Miamisburg, OH), and other reagents from Sigma-Aldrich Company (St. Louis, MO).

### Animals

180-200 g male Wistar rats were used. They were maintained in a 12h:12h light-dark cycle with free access to standard laboratory rat chow pellets (Panlab) and water. Animals were deprived of food 24 hours prior to hepatocyte isolation. Experiments were conducted according to guidelines accepted by the University Animal Care and Use Committee. Appropiate measures were taken to minimize pain or discomfort of animals.

### Preparation of cells and incubation

Suspensions of isolated parenchymal liver cells were prepared from 24-h starved animals as previously described [47]. Cells were resuspended in Krebs-Ringer bicarbonate buffer, pH 7.4. Preparations with viability below 90%, established by the trypan blue exclusion method, were not used. Samples (6 ml) of these suspensions, containing 2.3 × 10^6 ^cells/ml, were incubated at 37°C with gassing and continuous shaking (160 strokes/min, which is the minimum shaking that assures total suspension of cells) for 2 h, as it is the optimum time to ensure maximum glycogen synthesis without diminishing cell viability. Conditions for cell incubation were: a) 20 mM glucose, with glucose enriched 50% in [1,2-^13^C_2_]-glucose, b) 20 mM glucose + 9 mM lactate + 1 mM pyruvate (10 mM lactate/pyruvate (9:1)), with glucose enriched 50% in [1,2-^13^C_2_]-glucose and c) 20 mM glucose + 10 mM lactate/pyruvate (9:1), with lactate enriched 50% in [U-^13^C_3_]-lactate.

### Measurement of metabolites

At the beginning and end of incubations, cells were centrifuged (3000 g, 20 s), and incubation medium and cell pellets were obtained. For glycogen determination, cell pellets were immediately homogenized with 30% (w/v) KOH using a modification of Chan et al. methodology [48], where we have used 3 mM paper to precipitate glycogen. Glucose and lactate incubation medium concentrations were determined using spectrophotometric methods as described in [49,50].

### Gas Chromatography/Mass Spectrometry sample processing and analysis

At the end of incubations, cells were centrifuged so that the incubation medium and cell pellet are separated and everything was frozen in liquid nitrogen and stored at -80°C until processing for GC/MS analysis. Incubation media were processed for isolation of lactate, glucose, and glutamate using previously established methods [51,52]. Glycogen was isolated from cell pellets after ethanol precipitation of glycogen over 3 mM paper, and then treated with amyloglucosidase, and the hydrolyzed glucose was isolated using ion exchange chromatography [6]. Immediately after that, glucose from the medium or from hydrolyzed glycogen, as well as lactate and glutamate were derivatized for GC/MS analysis [51,53,54]. A mass selective detector HP 5973 equipment coupled to a gas chromatograph HP 6890 was used for all the metabolites as described elsewhere [51,53,54]. Chemical ionization was used to give the molecular ion (C1-C6) of the glycogen or medium glucose molecules at m/z 328, and the same for the lactate molecule (C1-C3) at m/z 328. Electron impact ionization was used to characterize isotopologues of C1-C4 (m/z 242) and C3-C6 (m/z 187) glycogen glucose fragments, as well as C2-C4 (m/z 152) and C2-C5 (m/z 198) glutamate fragments.

Results of the mass isotopologues in glucose, lactate and glutamate are reported as molar fractions of m0, m1, m2, etc, where m0, m1, m2... indicate the number of ^13^C atoms in the molecule [55]. The data for each independent experiment obtained after subtraction of natural ^13^C isotope enrichment are presented in Additional File 2.

## List of abbreviations

Metabolites: glc: glucose; glu: glutamate; lac: lactate; glgn: glycogen; g6p: glucose 6 phosphate; f6p: fructose 6-phosphate; fbp: fructose 1,6-bisphosphate; dhap: dihydroxyacetone phosphate; g3p: glyceraldehyde 3-phosphate; pep: phosphoenolpyruvate; pyr: pyruvate; accoa: acetyl coenzyme A; e4p: erythrose 4-phosphate; s7p: sedoheptulose 7-phosphate; r5p: ribose 5-phosphate; xu5p: xylulose 5-phosphate; mal: malate; oaa: oxaloacetic acid; cit: citrate. Enzymes: g6pase: glucose 6-phosphatase; gs: glycogen synthase; gp: glycogen phosphorylase; hk1 & hk2: hexokinase; fbp1 and fbp2: fructose 1,6-bisphosphatase; pfk1 and pfk2: phosphofructokinase; ald: aldolase; tk: transketolase; ta: transaldolase; g6pdh: glucose 6-phosphate dehydrogenase; pck: phosphoenolpyruvate carboxykinase; pk: pyruvate kinase; pdh: pyruvate dehydrogenase complex; cs: citrate synthase; pc: pyruvate carboxylase.

## Authors' contributions

IMM performed the analysis and wrote the paper, VAS developed the algorithms and wrote the paper, SM made the experiments and analyzed the data, JR analyzed the data, MO analyzed the data, LA analyzed the data and wrote the paper, MC analyzed the data and wrote the paper. All authors read and approved the final manuscript.

## Supplementary Material

Additional file 1 **Differential equations of the used kinetic models**. Kinetic models were used to simulate the total fluxes and concentrations of metabolites. Based on these calculated total values Isodyn further simulates the distribution of isotopic isomers. Click here for file

Additional file 2 **Measured distributions of ^13^C isotopomers in metabolites**. The table presents the data for each independent experiment obtained as described in Methods after subtraction natural ^13^C isotope enrichment. Click here for file
