# Supplementary material for: Compartmentation of glycogen metabolism revealed from 13C isotopologue distributions
Source: BMC Syst Biol. 2011 Oct 28;5:175. doi: 10.1186/1752-0509-5-175 (PMC3292525; doi:10.1186/1752-0509-5-175)
Supplement: Additional file 1 — Differential equations of the used kinetic models. Kinetic models were used to simulate the total fluxes and concentrations of metabolites. Based on these calculated total values Isodyn further simulates the distribution of isotopic isomers. [file 1752-0509-5-175-S1.PDF]

Differential equation corresponding to the model accounting a single well-mixed pool of hexose phosphates (model A).

1. Hexose phosphates (g6p and f6p) are combined in a single pool (h6p).Glycolitic pool is described as:

$$d(h6p)/dt=(hk1-pfk1+fbp1-g6pdh-h6pout-tk_{f6p5}+tk_{p5f6}-tk_{f6s7}+tk_{s7f6}-ta_{f6s7}+ta_{s7f6}-gsn)/Vi$$

2. fructose bisphosphate :

$$d(fbp)/dt=(pfk1-fbp1-ald)/Vi$$

3. trioses (glyceraldehyde-3-phosphate + dihydroxyacetone phosphate):

$$d(t3p)/dt=(2\cdot ald-g3pep+pepg3-g3out+tk_{p5s7}-tk_{s7p5}+tk_{p5f6}-tk_{f6p5}+ta_{f6s7}-ta_{s7f6})/Vi$$

4. phosphoenol pyruvate :

$$d(pep)/dt=(g3pep-pepg3-pk+pepck-pepout)/Vi$$

5. intracellular pyruvate and lactate are combined in a single pool (pyr):

$$d(pyr)/dt=(pk-pc-pdh-pyrout+pyrin+lacin-lacout)/Vi$$

6. oxaloacetate :

$$d(oaa)/dt=(pc+malooa-oamal-cs-pepck-oaout)/Vi$$

7. malate:

$$d(mal)/dt=(citmal-maloa+oamal)/Vi$$

8. citrate:

$$d(cit)/dt=(cs-citmal-citout+citin)/Vi$$

9. acetil CoA:

$$d(accoa)/dt=(pdh-cs-coaout+coain)/Vi$$

10. pentoses phosphate (xylulose-5-phosphate+ribose-5-phosphate):

$$d(p5p)/dt=(g6pdh-2\cdot tk_{p5s7}+2\cdot tk_{s7p5}+tk_{f6p5}-tk_{p5f6}-r5pout)/Vi$$

11. eritrose-4-phosphate :

$$d(e4p)/dt=(ta_{s7f6}-ta_{f6s7}+tk_{f6s7}-tk_{s7f6}+tk_{f6p5}-tk_{p5f6}-e4pout)/Vi$$

12. sedoheptulose-7-phosphate:

$$d(s7p)/dt=(tk_{p5s7}-tk_{s7p5}+tk_{f6s7}-tk_{s7f6}+ta_{s7f6}-ta_{f6s7}-s7pout)/Vi$$

Differential equation corresponding to the model accounting for channeling of hexose phosphates to glycogen (model B).

1. Hexose phosphates (g6p and f6p) are combined in a single pool (h6p).Glycolitic pool is described as:

$$d(h6p)/dt=(hk1-pfk1+fbp1-g6pdh-h6pout-tk_{f6p5}+tk_{p5f6}-tk_{f6s7}+tk_{s7f6}-ta_{f6s7}+ta_{s7f6})/Vi$$

2. hexoses channeled to glycogen:

$$d(h6p_c)/dt=(-pfk2+fbp2+hk2-gsn)/Vi$$

3. fructose bisphosphate :

$$d(fbp)/dt=(pfk1-fbp1-ald+pfk2-fbp2)/Vi$$

4. trioses (glyceraldehyde-3-phosphate + dihydroxyacetone phosphate):

$$d(t3p)/dt=(2\cdot ald-g3pep+pepg3-g3out+tk_{p5s7}-tk_{s7p5}+tk_{p5f6}-tk_{f6p5}+ta_{f6s7}-ta_{s7f6})/Vi$$

5. phosphoenol pyruvate :

$$d(pep)/dt=(g3pep-pepg3-pk+pepck-pepout)/Vi$$

6. intracellular pyruvate and lactate are combined in a single pool (pyr):

$$d(pyr)/dt=(pk-pc-pdh-pyrout+pyrin+lacin-lacout)/Vi$$

7. oxaloacetate :

$$d(oaa)/dt=(pc+malooa-oamal-cs-pepck-oaout)/Vi$$

8. malate:

$$d(mal)/dt=(citmal-maloa+oamal)/Vi$$

9. citrate:

$$d(cit)/dt=(cs-citmal-citout+citin)/Vi$$

10. acetil CoA:

$$d(accoa)/dt=(pdh-cs-coaout+coain)/Vi$$

11. pentoses phosphate (xylulose-5-phosphate+ribose-5-phosphate):

$$d(p5p)/dt=(g6pdh-2\cdot tk_{p5s7}+2\cdot tk_{s7p5}+tk_{f6p5}-tk_{p5f6}-r5pout)/Vi$$

12. eritrose-4-phosphate :

$$d(e4p)/dt=(ta_{s7f6}-ta_{f6s7}+tk_{f6s7}-tk_{s7f6}+tk_{f6p5}-tk_{p5f6}-e4pout)/Vi$$

13. sedoheptulose-7-phosphate:

$$d(s7p)/dt=(tk_{p5s7}-tk_{s7p5}+tk_{f6s7}-tk_{s7f6}+ta_{s7f6}-ta_{f6s7}-s7pout)/Vi$$

The terms in the differential equations listed above represent metabolic fluxes expressed as follows:

1. hk1: Hexokinase flux to glycolytic pool of hexoses

$$hk1=Frhk*Vhk1$$

Where:
Vhk1: flux of glucose uptake calculated from experimental data.
Frhk: fraction of glucose entrance directed to glycolysis

2. hk2: glucose flux directed to to glycogen

$$hk2=(1-Frhk)*Vhk1$$

3. g6pase1: Glucose-6-phosphatase flux from glycolytic pool of hexoses

$$g6pase1=g6p*Vg6p/(g6p+Kg6p)$$

Where:
Vg6p: hexose-6-phosphatase1, Vmax
Kg6p: hexose-6-phosphatase, Km

g6p: glucose 6 phosphate concentration:

4. g6pase2: Glucose-6-phosphate flux from glycogen production pool of hexoses
   $g6pase2 = h6pa * Vh6pII/(h6p_c + Kg6p)$
   Where:
      Vh6pII: hexose-6-phosphatase2 Vmax
      $h6p_c$: hexoses from glycogen production pool concentration
5. pfk1: phosphofructo kinase flux from glycolytic pool of hexoses
   $pfk1 = VpfkI * f6p/(f6p + Kf6p)$
   Where:
      VpfkI: phosphofructo kinase I Vmax
      Kf6p: phosphofructo kinase Km
6. pfk2: phosphofructo kinase flux from glycogen production pool of hexoses
   $pfk2 = VpfkII * h6p_c/(h6p_c + Kf6p)$
   Where:
      VpfkII: phosphofructo kinase II Vmax
7. fbpase1: fructose-bis-phosphatase flux to glycolytic pool of hexoses
   $fbpase1 = Frfbp * VfbpI * fbp/(fbp + Kfbp)$
   Where:
      Frfbp: fraction of fructose-bis-phosphatase activity
      VfbpI: fructose1,6 bisphosphatase Vmax
      Kfbp: fructose-bis-phosphatase Km
      fbp: fructose bis phosphate concentration
8. fbpase2: fructose-bis-phosphatase flux to glycogen production pool of hexoses
   $fbpase2 = (1 - Frfbp) * VfbpI * (fbp)/(fbp + Kfbp)$
9. gp: glycogen phosphorilase flux
   $gp = glgn * Vglgph/(glgn + Kglgph)$
   Where:
      Vglgph: glycogen phosphatase Vmax
      Kglgph: glycogen phosphatase Km
      glgn: glycogen concentration
10. gsn: glycogen syntase flux
    gsn = Vglgsyn
    Where:
       Vglgsyn; glycogen syntase Vmax
11. aldf: aldolase global forward flux
12. aldr:aldolase global reward flux
13. aldex: aldolase exchange flux between fructose bis-phosphate and glyceraldehyde-3-phosphate
14. g3pep: gliceraldhid phospahte deshidrogenase forward flux
    $g3pep = VGAPDHf * g3p/(g3p + KGAPDH)$
    Where:
       VGAPDHf: Glyceraldehyde 3-phosphate dehydrogenase Vmax
       KGAPDH: Glyceraldehyde 3-phosphate dehydrogenase Km
       g3p: glyceraldehyde concentration:
          $g3p = t3p/23$
          Where:
             t3p: trioses concentration
15. pepg3: gliceraldhid phospahte deshidrogenase reward flux
    $pepg3 = Vpep * pep/(pep + Kpep)$
    Where:
       Vpep: phosphoenol pyruvate Vmax

Kpep: phosphoenol pyruvate Km
pep: phosphoenol pyruvate concentration

16. pk: pyruvate kinase flux

$$pk=(VPK*pep/KPK)/(1+pep/KPK)$$

Where:
VPK: pyruvate kinase Vmax
KPK: pyruvate kinase Km
pep: phosphoenol pyruvate concentration

17. lacin: lactate deshidrogenase reward flux

$$lacin=lac*Vlacin/(Klacin+lac)$$

Where:
Vlacin: lactate in Vmax
Klacin: lactate in Km
lac: lactate concentration:

$$lac=0.89483*npyr$$

Where:
npyr: pyruvate and lactate in fast equilibrium

18. lacou: lactate deshidrogenase forward flux

$$lacout=lac*Vlaco/(Klaco+lac)$$

Where:
Vlaco: lactate out Vmax
Klaco: lactate out Km

19. pc: pyrivate carboxylase

$$pc=A*Vpc*pyr/(pyr+Kpyr)*(0.1+coa)/0.1$$

Where:
A: coheficient for pepck and pc
Vpc: pyruvate carboxilase Vmax
Kpyr: pyruvate carboxilase Km
coa: acetil CoA concentration
pyr: pyruvate concentration:

$$pyr=npyr-lac$$

20. pepck: phosphoenol-pyruvate kinase

$$pepck=A*Vpepck*oaa/(oaa+Kpepck)$$

Where:
Vpepck: phosphoenol pyruvate kinase Vmax
Kpepck: phosphoenol pyruvate kinase Km
oaa: oxalacetate concentration

21. maloa: malate dehydrogenase forward flux

$$maloa=B*Vmalooa*mal/(mal+Kmalooa)$$

Where:
B: coheficient for ooamal and malooa
Vmalooa: malate dehydrogenase forward Vmax
Kmalooa: malate dehydrogenase forward Km
mal: malate concentration

22. oamal: malate dehydrogenase reward flux

$$oamal=B*Vooamal*noaa/(noaa+Kooamal)$$

Where:
Vooamal: malate dehydrogenase reward Vmax
Kooamal: malate dehydrogenase reward Km
oaa: oxalacetate concentration

23. cs: citrate synthase

$$cs=D*VCS*oaa/(oaa+KCS)*(coa)/(coa+KCS)$$

Where:

D: coefficient for krebs cycle reactions

VCS: citrate syntase Vmax

KCS: citrate syntase Km

24. citmal: iso-citrate dehydrogenase flux

$$citmal = D * Viso * cit / (cit + Kiso)$$

Where:

Viso: isocitrate Vmax

Kiso: isocitrate Km

cit: citrate concentratio

25. pdh: pyruvate deshydrogenase flux

$$pdh = D * Vpdh * pyr / (pyr + Kpdh) * (0.1 + oaa) / 0.1$$

Where:

Vpdh: pyruvate deshidrogenase Vmax

Kpdh: pyruvate deshidrogenase Km

26. g6pdh: pentose phosphatase pathway flux

$$g6pdh = (Vppp * g6p) / (g6p + Kppp)$$

Where:

Vppp: pentoses phosphate pathway Vmax

Kppp: pentoses phosphate pathway Km

27. tk_{p5s7}: transketolase flux between pentose-5-phosphate ↔ sedoheptulose-7-phosphate
28. tk_{s7p5}: transketolase flux between sedoheptulose-7-phosphate ↔ ribose-5-phosphate
29. tk_{p5f6}: transketolase flux between fructose-6-phosphate ↔ pentose-5-phosphate
30. tk_{f6p5}: transketolase flux between pentose-5-phosphate ↔ fructose-6-phosphate
31. tk_{f6s7}: transketolase flux between fructose-6-phosphate ↔ sedoheptulose-7-phosphate
32. tk_{s7f6}: transketolase flux between sedoheptulose-7-phosphate ↔ fructose-6-phosphate
33. tk_{p5g3}: transketolase flux between xilulose-5-phosphate ↔ glyceraldehyde-3-phosphate
34. ta_{f6s7}: transaldolase flux between fructose-6-phosphate ↔ sedoheptulose-7-phosphate
35. ta_{s7f6}: transaldolase flux between sedoheptulose-7-phosphate ↔ fructose-6-phosphate
36. ta_{f6g3}: transaldolase flux between fructose-6-phosphate ↔ glyceraldehyde-3-phosphate
37. ta_{s7e4}: transaldolase flux between sedoheptulose-7-phosphate ↔ eritrose-4-phosphate
38. f6pout: outflow of fructose-bis-phosphatase

$$f6pout = Vf6po * f6p / (f6p + Kf6po)$$

Where:

Vf6po: fructose-6-phospahte out Vmax

Kf6po: fructose-6-phospahte out Km

39. pyr: outflow of pyruvate

$$pyrout = Vpyro * pyr / (pyr + Kpyro)$$

Where:

Vpyro: pyruvate out Vmax

Kpyro: pyruvate out Km

40. pepout: outflow of phosphoenol-pyruvate

$$pepout = Vpepo * pep / (pep + Kpepo)$$

Where:

Vpepo: phosphoenol pyruvate out Vmax

Kpepo: phosphoenol pyruvate out Km

41. g3out: outflow of trioses phosphate

$$g3out = Vdhapo * t3p / (t3p + Kdhapo)$$

Vdhapo: trioses phosphate out Vmax

Kdhapo: trioses phosphate out Km

42. pyrin: inflow of pyruvate

$$pyrin = Vpyrin$$

Where:

Vpyrin: pyruvate in flux Velocity

43. oaaout: outflow of oxalacetate

$oaaout = Vooao * oaa/(oaa + Kooao)$

Where:

Vooao: oxalacetate out Vmax

Kooao: oxalacetate out Km

44. citout: outflow of citrate

$citout = Vcito * cit/(cit + Kcito)$

Where:

Vcito: citrate out Vmax

Kcito: citrate out Km

45. citin: inflow of citrate

$citin = Vcitin$

Where:

Vcitin: citrate in flux Velocity

46. coaout: outflow of acetil CoA flux

$coaout = Vcoao * coa/(coa + Kcoao)$

Where:

Vcoao: acetil CoA out Vmax

Kcoao: acetil CoA out Km

47. coain: inflow of acetil CoA

$coain = Vcoain$

Where:

Vcoain: acetil CoA in flux Velocity

48. r5pout: outflow of ribose-5-phosphate

$r5pout = Vr5o * r5p/(r5p + Kr5o)$

Where:

Vr5o: pentoses out Vmax

Kr5o: pentoses out Km

r5p: ribose-5-phosphate:

$r5p = p5p - xu5p$

Where:

p5p: pentoses phosphate

xu5p: xilulose-5-phosphate:

$xu5p = (p5p * keq)/(1 + keq)$

Where:

$keq = 1.24$

49. e4pout: outflow of eritrose-4-phosphate

$e4pout = Ve4po * e4p/(e4p + ke4po)$

Where:

Ve4po: eritrose-4-phosphate out Vmax

ke4po: eritrose-4-phosphate out constant rate

e4p: eritrose-4-phosphate

50. s7pout: outflow of sedoheptulose-7-phosphate

$s7pout = Vs7po * s7p/(s7p + Ks7po)$

Where:

Vs7po: sedoheptulose-7-phosphate out Vmax

Ks7po: sedoheptulose-7-phosphate out constant rate

s7p: sedoheptulose-7-phosphate

The fluxes from 11 to 13 that corresponds to aldolase activity are described in present paper

The fluxes from 27 to 33that corresponds to transketolase and the fluxes from 34 to 38 that corresponds to transaldolase were previously described in [12]
